# Glioblastoma cell motility and invasion is regulated by membrane-associated heat shock protein Hsp70

**DOI:** 10.1007/s11060-025-05127-5

**Published:** 2025-06-24

**Authors:** Ruslana Likhomanova, Elena Oganesyan, Natalia Yudintceva, Georgii Fofanov, Anastasiia Nechaeva, Alexei Ulitin, Aleksander Kim, Nikolay Aksenov, Alla Shatrova, Rustam Ziganshin, Danila Bobkov, Konstantin Samochernykh, Stephanie E. Combs, Maxim Shevtsov

**Affiliations:** 1 Institute of Cytology of the Russian Academy of Sciences (RAS), St. Petersburg, Russia; 2 Personalized Medicine Centre, Almazov National Medical Research Centre, St. Petersburg, Russia; 3 Polenov Neurosurgical Institute, Almazov National Medical Research Centre, St. Petersburg, Russia; 4 Shemyakin-Ovchinnikov Institute of Bioorganic Chemistry Russian Academy of Sciences (RAS), Moscow, Russia; 5 Klinikum Rechts der Isar, Technical University of Munich, Munich, Germany

**Keywords:** Membrane-associated Hsp70, Glioblastoma multiforme, Migration, Invasion

## Abstract

**Purpose:**

Membrane-associated heat shock protein 70 kDa (mHsp70) is specifically localized on the plasma membrane of various malignant tumor cells. In current study, we investigated the role of mHsp70 in motility of glioblastoma cells, which are known to be characterized by a high ability to migrate and invade surrounding brain tissue.

**Methods:**

The presence of mHsp70 on the membrane of patient-derived glioblastoma cells was detected with confocal microscopy, flow cytometry, Western blot, and proteome analysis of lipid rafts. The motility and invasion characteristics were studied using automatic single-cell tracking and transwell analysis with Hsp70 inhibitors.

**Results:**

mHsp70 is able to influence the migration and invasion of glioblastoma cells, and the degree of protein expression correlates with motility. The involvement of mHsp70 in the regulation of cell motility is likely to be mediated by interactions with proteins responsible for cytoskeletal remodeling and connection with the extracellular matrix. Moreover, the protein is localized in lipid rafts associating with other members of the HSPs families. The application of small molecule Hsp70 inhibitors PES and JG-98 successfully reduced the migratory and invasive potential, which allowed them to be used as an adjuvant agent in tumor therapy.

**Conclusion:**

This study expands our understanding of mHsp70 function in cancer cells and contributes to the development of novel approaches to the treatment of malignant tumors.

**Supplementary Information:**

The online version contains supplementary material available at 10.1007/s11060-025-05127-5.

## Introduction

Glioblastoma (GBM) is the most common and aggressive form of primary brain tumors in adults. Despite the enormous progress in surgical methods and the development of therapeutic agents, the average life expectancy of patients with GBM does not exceed 15 months with a standard form of treatment combining surgical resection and subsequent radiation and chemotherapy with temozolomide [1]. One of the key factors complicating treatment is the highly infiltrative and invasive nature of the tumor, as well as the extremely increased migration potential of GBM into the surrounding healthy parenchyma of the brain [2, 3].

Stress-inducible heat shock protein 70 kDa (Hsp70) is one of the promising prognostic markers of malignant tumors, particularly for GBM [4, 5]. Besides its intracellular localization, Hsp70 is exposed on the surface of the plasma membrane of cancer cells, but not on normal cells, making it an attractive target for theranostics [6]. Positive expression of membrane-associated Hsp70 (mHsp70) has been found not only in a wide variety of primary tumors, but also in distant metastases [7, 8]. The specific presence of the chaperone in the membrane may be due to its electrostatic and hydrophobic interactions with molecules contained in lipid rafts (ceramide Gb3) [9], as well as lipids such as phosphatidylserine [10–12]. In this study we investigated the role of the chaperone in the migration and invasion of patient-derived glioblastoma cells. Indeed, Hsp70 was recently found to positively regulate TGF-α-induced migration of Huh-7 hepatocellular carcinoma cells via the AKT signaling pathway [13]. In head and neck squamous cell carcinoma tissues, Hsp70 expression correlates with the expression of proteins associated with the formation of invadopodia (HIF1-α, cortactin, MMP2, MMP14) [14]. However, these studies consider all cellular Hsp70, without distinguishing it by localization– cytoplasmic, nuclear or membrane.

In our previous study, we confirmed that GBM tissue is positive for the mHsp70 compared to healthy brain tissue, and the membrane chaperone correlates with the migratory activity of malignant brain tumor cell lines in vitro [15]. Continuing our hypothesis, we investigated the role of mHsp70 in the migration and invasion of glioblastoma cells using the model of unique primary tumor cells obtained from intraoperative material of neuro-oncological patients. We showed significant mHsp70 expression in primary cells and a positive association between the mHsp70 and the cells’ migratory/invasive properties. This highlights its essential role in regulating tumor cell motility. To further implicate the role of the membrane-bound chaperone in cell motility we have employed inhibitors Hsp70 PES and JG-98, demonstrating their potential for the treatment of malignant brain tumors.

## Materials and methods

### Patients

The study was performed using primary human glioblastoma cell cultures isolated from intraoperative biopsy material of adult or pediatric patients diagnosed with GBM provided by the Russian Scientific Research Neurosurgical Institute named after A. L. Polenov and Almazov Medical Research Center. The study protocol was approved by the Ethics Committee of the Almazov Medical Research Center (resolution No. 2712-20 dated December 21, 2020). All patients received informed written consent to participate in the study. We confirm that all experiments have been conducted in accordance with the relevant guidelines and rules. Characteristics of patients presented in Table S1.

### Cells

Primary glioblastoma cells (coined the abbreviations ANI, GSN, IBD, TMS) were obtained from fragments of intraoperative material from patients newly diagnosed with GBM (grade 4) without prior treatment. The cells were isolated using a three-dimensional explant model and cultured at 37 °C in a 5% CO_2_ in DMEM/F12 medium (Thermo Fisher Scientific, Waltham, Massachusetts, USA) containing B27 without vitamin A (50X, Thermo Fisher Scientific, Waltham, Massachusetts, USA), EGF and bFGF (20 ng/mL, PSG130-100, PSG060-100, SCI store, Moscow, Russia). For experiments, a suspension of single cells was obtained using Accutase solution (400 units/ml, Sigma-Aldrich, Burlington, Massachusetts, USA).

### Confocal microscopy

The expression of mHsp70 in tumor cells was visualized by the confocal system Olympus FV3000 (Olympus, Tokyo, Japan). Cells on glass slides were incubated with FITC-mHsp70 monoclonal antibody (mAb) (SPA810, StressMarq Biosciences Inc, Victoria, Canada) for 25 min on ice in the dark. After incubation, cells were washed, fixed and mounted in Mounting Medium with 4′,6-diamidino-2-phenylindole (DAPI, 50011, Ibidi, Gräfelfing, Germany). The cells were also stained with III β-tubulin mAb (2G10, MA1-118, Thermo Fisher Scientific, Waltham, Massachusetts, USA), GFAP polyclonal antibody (PA5-16291, Thermo Fisher Scientific, Waltham, Massachusetts, USA), eFluor 570-SOX2 mAb (Btjce, 41-9811-82, Thermo Fisher Scientific, Waltham, Massachusetts, USA), and Alexa Fluor 647-Nestin mAb (10C2, 656810, BioLegend, San Diego, California, USA).

### Flow cytometry and FACS

Analysis of the surface mHsp70 and CD133 expression in primary cells was performed by the CytoFLEX flow cytometer (Beckman Coulter, Brea, California, USA). Cell suspension was stained with FITC-mHsp70 mAb (SPA810, StressMarq Biosciences Inc, Victoria, Canada) or PE-CD133 mAb (293C3, ab253271, Abcam, USA) for 25 min on ice in the dark. Unstained cells and cells incubated with FITC-conjugated IgG1 mAb (679.1Mc7, A07795, Beckman Coulter, Brea, California, USA) or PE-conjugated IgG2 mAb (E5Y6Q, 62937, Cell Signaling Technology, Danvers, Massachusetts, USA) were used as a control. Based on the cytograms obtained after staining for mHsp70 each primary cell cultures were sorted into two subpopulations – high expressed mHsp70 (mHsp70^High^) and low expressed mHsp70 (mHsp70^Low^). Fluorescence activated cell sorting (FACS) was performed on S3e Cell Sorter (Bio-Rad, Hercules, California, USA). The quantitative content of subpopulations based on the gating results is presented in Table S2. Before using Hsp70 inhibitors in motility assays, their effect on cell viability was pre-tested using propidium iodide (PI, G1021-10ML, ServiceBio, Wuhan, China) staining.

### Western blot

To confirm the presence of Hsp70 on the membrane, fractions (membrane and cytoplasmic) were isolated from ANI, GSN, IBD, and TMS cells using the Mem-PER™ Plus Membrane Protein Extraction Kit (89842, Thermo Fisher Scientific, Waltham, Massachusetts, USA) according to the manufacturer’s instructions. The obtained fractions were analyzed by Western blot. The samples were separated using 12% polyacrylamide gel electrophoresis and transferred to a nitrocellulose membrane (1620112, Bio-Rad, Hercules, California, USA). The proteins were incubated with primary mAb to Hsp70 (1:1000, EPR16892, ab181606, Abcam, Cambridge, UK) and visualized using the SuperSignal™ West Femto Maximum Sensitivity Substrate chemiluminescence reaction kit (34094, Thermo Fisher Scientific, Waltham, Massachusetts, USA) on ChemiDoc Touch (Bio-Rad, Hercules, California, USA).

### Isolation of lipid rafts

Lipid rafts were isolated from 15 × 10^6^ ANI, GSN, IBD, and TMS cells by nonionic detergent lysis and sucrose gradient ultracentrifugation as described in [16]. For subsequent mass spectrometric analysis, hydrolysis and trypsinolysis of the isolated proteins were performed. The peptides were then desalted on an Empore SDB-RPS microcolumn (3 M, Two Harbors, Minnesota, USA) [17], washed sequentially with solvent mixtures containing trifluoroacetic acid/ethyl acetate, and dried.

### HPLC-MS/MS analysis

Samples were separated by reversed-phase chromatography using an Ultimate 3000 Nano LC System (Thermo Fisher Scientific, Waltham, Massachusetts, USA) coupled to an Orbitrap Lumos Tribrid mass spectrometer (Thermo Fisher Scientific, Waltham, Massachusetts, USA) via a nanoelectrospray source (Thermo Fisher Scientific, Waltham, Massachusetts, USA). The samples were applied to a pre-column (50 × 0.1 mm) with Reprosil-Pur 200 C18-AQ 5 m sorbent (Dr. Maisch GmbH, Tübingen, Germany) and separated at room temperature on a fused silica column (300 × 0.1 mm) with an P2000 Laser Puller (Sutter, Novato, California, USA). Mass spectrometric analysis was performed with the following settings: MS1 scan: resolution 60,000, scan range 350–1600 m/z, maximum ion injection time auto, AGC level standard, MS2 scan: resolution 15,000, HCD fragmentation with energy 30%, maximum ion injection time 80 ms, AGC level standard. The obtained mass spectrometric data were analyzed using Peaks studio 10.0 software (Bioinformatics Solutions Inc., Waterloo, Canada). Protein identification was performed by searching for correlation of mass spectra with the Uniprot SwissProt human protein sequence database with the following parameters: constant Cys modification – carbamidomethylation, variable modifications – Asn/Gln deamidation, Met oxidation and N-terminal amino group acetylation, acceptable level of false positive peptide identifications – 0.01, protease specificity – C-terminus of Arg and Lys. The deviation of the experimental mass of the peptide from the theoretical mass was allowed to be up to 10 ppm, and the deviation of the fragments mass was allowed to be up to 0.05 Da. The list of identified proteins was loaded into Cytoscape v3.10.2 platform, equipped with access to the STRING online protein search tool. The built-in yfiles-organic and remove overlaps layout algorithms were applied to the resulting network, and a network of neighborhood Hsp70 (HSPA1B) interactors was obtained.

### Transwell analysis

The transwell method was used to assess tumor cells invasion characteristics. The directed movement of cells in a Transwell chamber (NEST, Wuxi, Jiangsu, China) through a permeable PET-membrane (pore diameter 3.0 μm) is driven by a concentration gradient of fetal bovine serum (FBS; HyClone, Tauranga, New Zealand). A matrigel solution (356234, Corning, New York, USA) was placed in the upper chamber before polymerization, and the remnants of the unpolymerized matrigel were removed. Then, a suspension of cells without FBS was placed in the upper chamber of the Transwell system, and a culture medium with 10% FBS was placed in the lower chamber. Additionally, Hsp70 inhibitors were added to the cell suspension: 1 µM 2-phenylethylsulfonamide/pifithrin-µ (PES; 506155, Sigma-Aldrich, Burlington, Massachusetts, USA), which binds to the substrate-binding domain of Hsp70, or 50 nM benzothiazole-acyanin (JG-98; HY-117282, MedChemExpress, Monmouth Junction, New Jersey, USA), which interacts with the nucleotide-binding domain. *Native populations of ANI*,* GSN*,* IBD*,* TMS cells were used as controls.* The concentrations are based on their preliminary testing on rat glioma C6, human glioblastoma T98G and U251 cell lines [15]. After 48 h, the membrane was fixed and stained with a 1% crystal violet solution (ServiceBio, Wuhan, China). Cells that did not pass through the membrane were removed with a cotton swab. 10 random non-overlapping fields of view were captured on each membrane (*n* = 3) using inverted microscope Nikon Eclipse TS100 (Nikon, Tokyo, Japan). *The invasive capacity of native untreated cells was taken as 100%*.

### Automatic single-cell tracking

The automatic imaging system Image ExFluorer (LCI, Namyangju-si, Gyeonggi-do, Korea) was used to determine the motility characteristics of mHsp70^High^ and mHsp70^Low^ glioblastoma cell subpopulations. Cells were cultured in a plate pre-coated with matrigel solution (356234, Corning, New York, USA). The cell nuclei were stained with Hoechst 33,342 (R37165, Thermo Fisher Scientific, Waltham, Massachusetts, USA). Intravital imaging of cells (*n* ≥ 750) was carried out over 24 h, taking frames every 15 min. Hsp70 inhibitors PES (1 µM) or JG-98 (50 nM) were added to the cells immediately before the experiment. Native unsorted cell subpopulations (mHsp70^Wt^) were used as controls. The captured images were analyzed using NIS-Elements software with a module for automatic segmentation, quantification and tracking of individual cells: cell nuclei were recognized in the images and the coordinates of the nuclei along the X-Y axis were recorded, cell movement tracks were constructed, the mean speed and straightness of track were calculated.

### Statistical processing

Statistical processing of the obtained data was carried out using GraphPad Prism 10.2.3 software (GraphPad Software Inc., California, USA). All experiments had at least three independent replicates. The data is presented as median with 95% confidence intervals (CI). The normality of data was performed using Kolmogorov-Smirnov and Shapiro-Wilk tests. All the data was distributed abnormally. The Mann-Whitney t-test was used to analyze the differences compared to the control group. The differences were considered statistically significant at *P* < 0.05. Statistical analysis specifics are presented in the Supplementary Information.

## Results

### Primary glioblastoma cells are positive for mHsp70

**Fig. 1:**
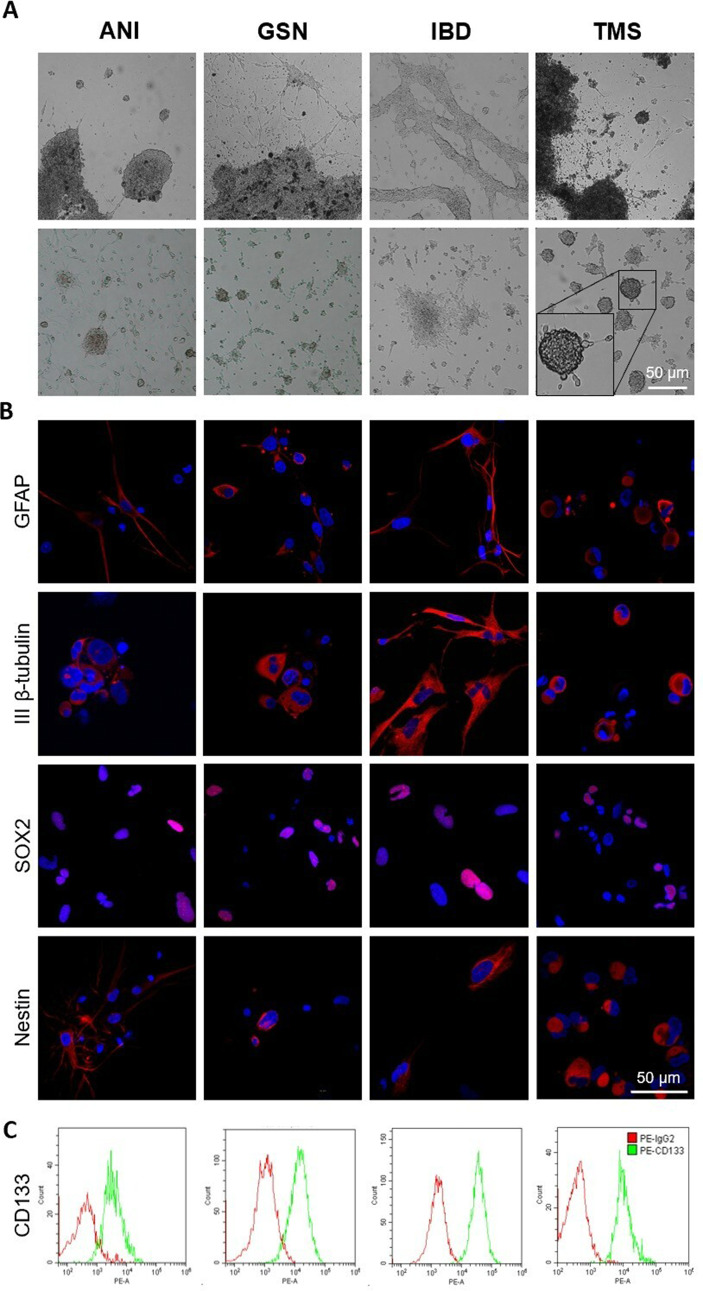
Characteristics of primary cultures of glioblastoma cells ANI, GSN, IBD, TMS. (**A**) Light microscopy photos showing cell morphology and an example of cell migration from an explant, scale bar 50 μm. (**B**) Confocal microscopy images detecting expression of GFAP (red), III β-tubulin (red), SOX2 (red), and Nestin (red). DAPI was used to stain the nuclei (blue), scale bar 50 μm. (**C**) Flow cytometry analysis of glioblastoma cells staining with PE-CD133 mAb (green curve), the PE-IgG2 mAb (red curve) was used as a control

Four primary cultures of glioblastoma cells were obtained from the intraoperative material of patients with newly diagnosed GBM, using a three-dimensional explant culture (Fig. 1A). Following 2–3 months of cultivation, the cells began to radially migrate from a piece of tumor and spontaneously form spheroids of various diameters (from 200 μm to 2 mm). Spheroids could also attach to the plate, after single cells migrate from them and independently form new ones. To confirm the cell origin, we showed that primary glioblastoma cultures express intermediate filament GFAP and microtubule III β-tubulin using confocal microscopy (Fig. 1B). The cells were also positive in stem markers including nuclear SOX2, cytoplasmic Nestin, and surface CD133 (Fig. 1B, C).

**Fig. 2:**
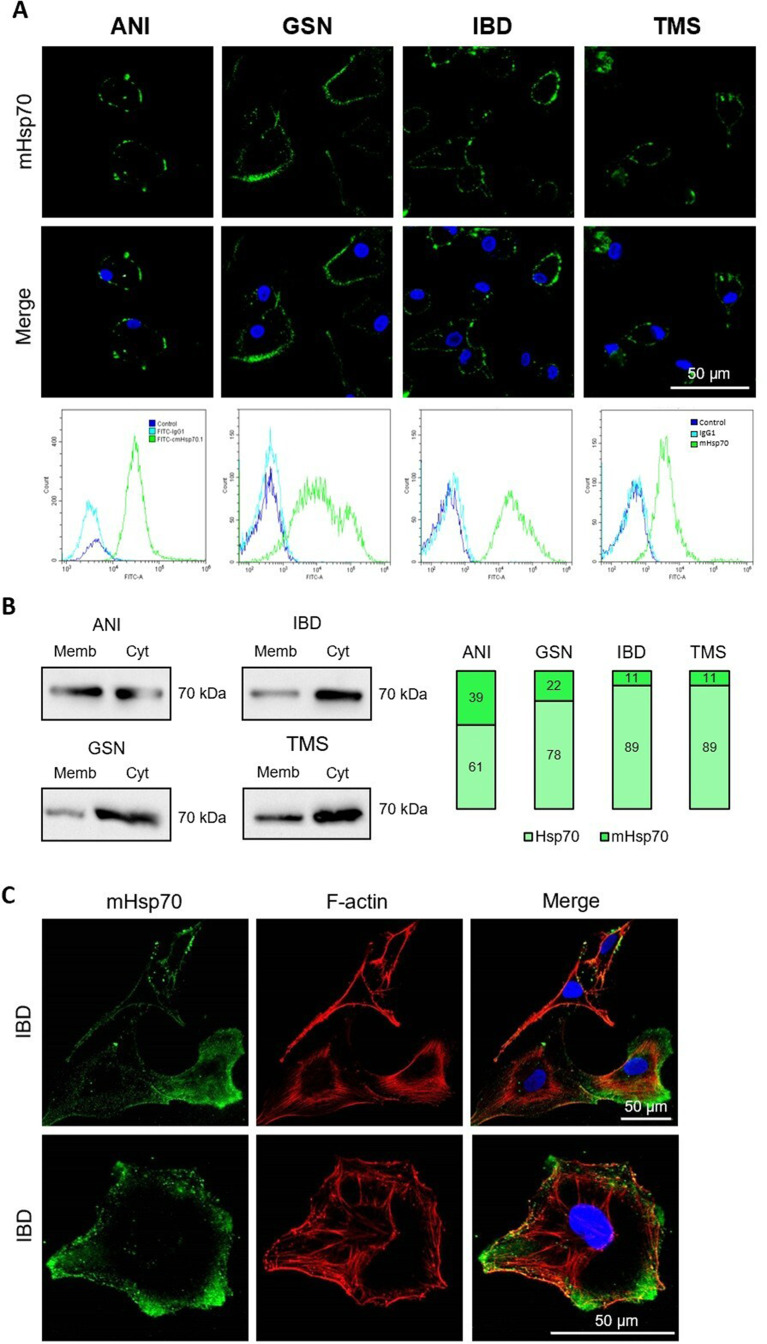
Primary glioblastoma cells express mHsp70 on their surface. (**A**) Confocal microscopy images and flow cytometry diagram of ANI, GSN, IBD, TMS cells; mHsp70 was detected with FITC-conjugated Hsp70 mAb (green), nuclei were detected with DAPI (blue), scale bar 50 μm. (**B**) Western blot of Hsp70 in membrane and cytoplasmic cell fractions. Plots show the percentage of Hsp70 in fractions normalized to the total amount of cytoplasmic Hsp70. (**C**) Confocal microscopy images of IBD cells stained for mHsp70 (green) and F-actin (red), nuclei were detected with DAPI (blue); scale bar 50 μm

The results of confocal microscopy, flow cytometry and Western blot of membrane fractions indicated the cells expressed mHsp70 on their surface, moreover staining was observed in 100% of the cell populations (Fig. 2A and B). We also analyzed the ratio of membrane to cytoplasmic forms of the protein (%) by Western blot using ImageJ software (NIH, Washington, USA). ANI cells were characterized by the highest mHsp70 content compared to other primary cultures. Interestingly, the protein was localized predominantly in the region of lamellipodia and filopodia, which are the key structures for ensuring cell migration and adhesion to the substrate (Fig. 2C).

### mHsp70 interacts with proteins regulating cell motility

**Fig. 3:**
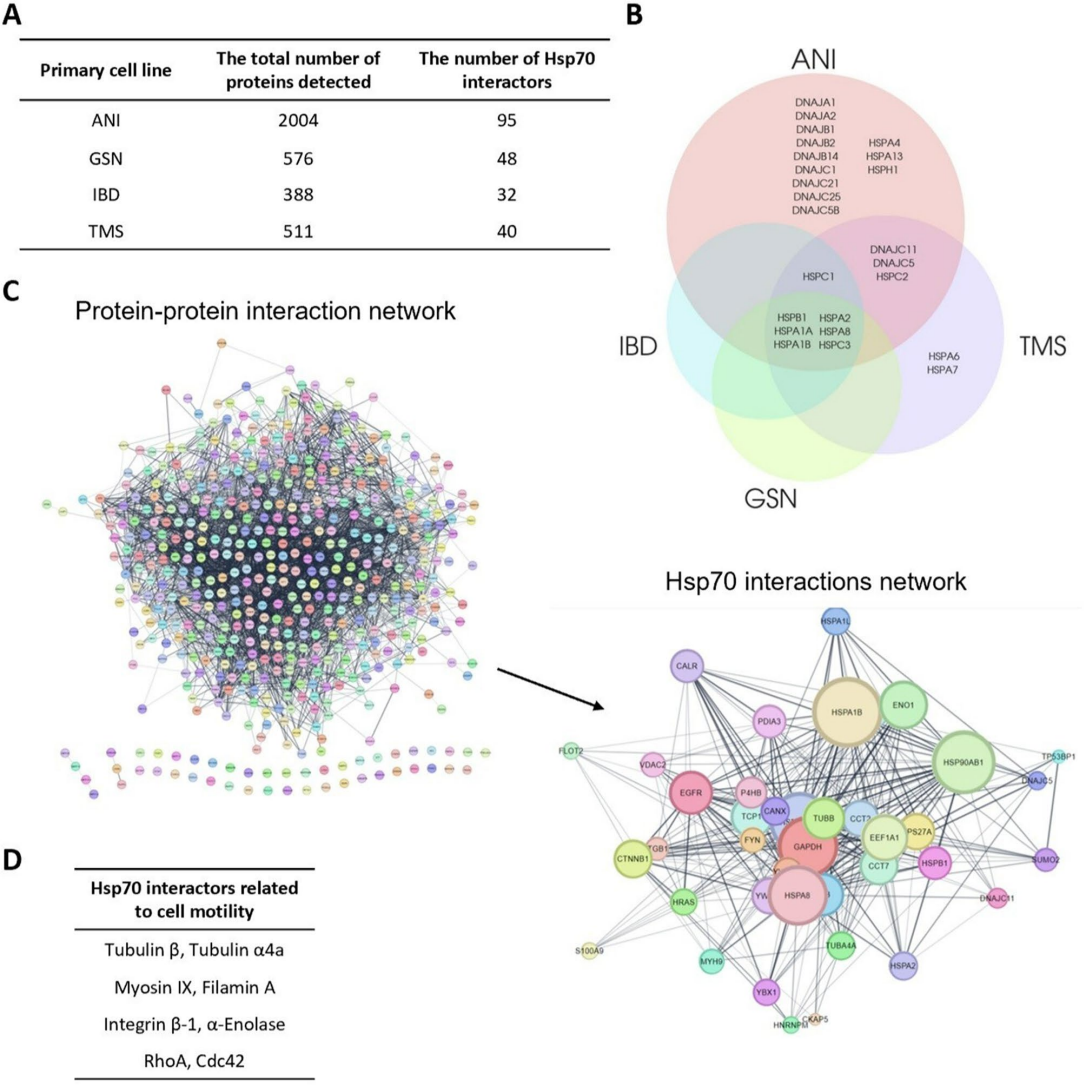
Proteome analysis of lipid rafts of primary glioblastoma cells ANI, GSN, IBD, TMS. (**A**) Quantitative information on the total content of raft-associated proteins and Hsp70 interactors. (**B**) Venn diagram of membrane chaperone clusters found in four cell lines. (**C**) Representative network of protein-protein interactions made for TMS cells and Hsp70 interactions network. (**D**) Hsp70 has a direct relationship with the listed proteins involved in cytoskeleton remodeling, cell adhesion and migration

mHsp70 is thought to localize to lipid rafts of tumor cells, which are rich in cholesterol and serve as а platform for signal transduction [18]. In this regard, we isolated lipid raft-enriched membrane regions from primary cells and performed their HPLC-MS/MS analysis. The results of proteomic analysis revealed a large number of proteins located in ordered domains of the tumor cell membrane, including a stress-inducible Hsp70 (Fig. 3A). Moreover, it turned out that the cell membrane is enriched with a whole cluster of chaperones, including not only Hsp70, but also other members of the families: the small HSPs, HSP40, HSP70, HSP90, and HSP110 (Fig. 3B). Making general protein-protein interaction networks allowed us to identify which proteins Hsp70 has a direct relationship with and who its possible interactors are. In addition to co-chaperones, we found strong association with glyceraldehyde-3-phosphate dehydrogenase (GAPDH), epidermal growth factor receptor (EGFR), β-catenin, flotillin-2, etc. Intriguingly, among the proteins interacting with Hsp70, there were a large number of proteins involved in cytoskeletal remodeling, cell adhesion and migration (Fig. 3C). These are tubulin-β, tubulin-α4a, myosin IX, actin-binding protein 2, filamin A, plakoglobin, integrin β-1, α-enolase and the small GTPase RhoA (Fig. 3D). The interaction of mHsp70 with these proteins may indicate the involvement of the chaperone in the regulation of migration and invasion of malignant brain tumor cells.

### mHsp70 regulates the migration and invasion ability of glioblastoma cells

**Fig. 4:**
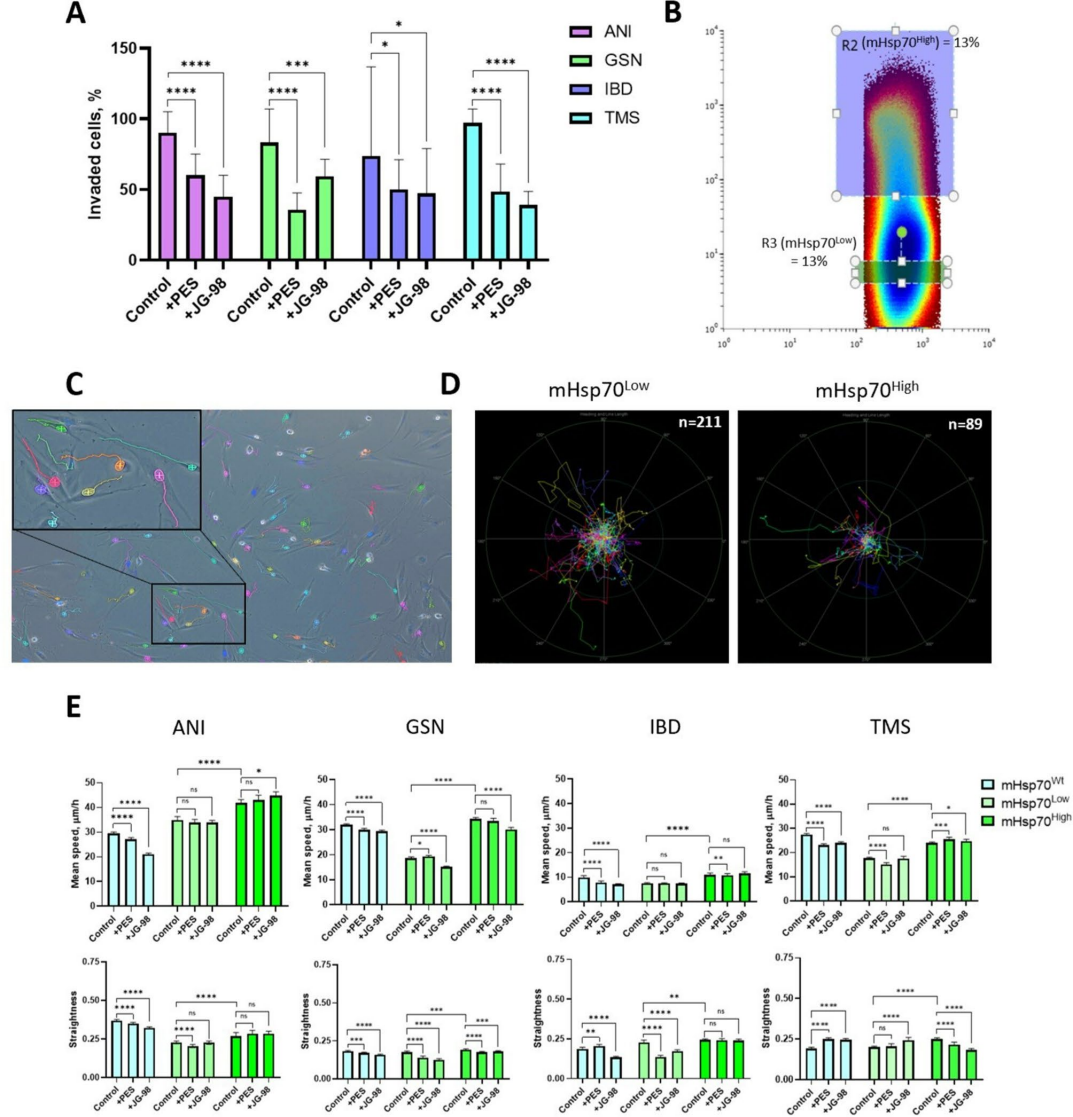
mHsp70 is involved in the regulation of migration and invasion of primary glioblastoma cells ANI, GSN, IBD, TMS. (**A**) Results of transwell assay of cells invading through a pre-coated porous membrane and treated with Hsp70 inhibitors PES (1 µM) or JG-98 (50 nM). At least 60 fields of view were analyzed in each group. Data are presented as median ± 95% CI. *The invasive capacity of native untreated cells was taken as 100%*. The statistical significance is shown as *p ≤ 0.05, ***p ≤ 0.001, ****p ≤ 0.0001. (**B**) Example of gating cells into mHsp70^Low^ and mHsp70^High^ subpopulations using FACS. (**C**) Example of cell movement tracks. (**D**) Example of rose plots with normalized cell movement tracks of mHsp70^Low^ and mHsp70^High^ subpopulations. (**E**) Results of single-cell tracking characterized by mean speed (µm/h) and straightness of trajectory. At least 750 cells were analyzed in one group. Data are presented as median ± 95% CI. The statistical significance is shown as *p ≤ 0.05, ***p ≤ 0.001, and ****p ≤ 0.0001, *ns* - not significant

To confirm the possible involvement of mHsp70 in glioblastoma cell invasion, we performed a transwell assay using Hsp70 inhibitors. The inhibitors PES, which blocks the activity of the substrate-binding domain of the protein, and JG-98, which connects with the nucleotide-binding domain, were used at non-toxic concentrations, as demonstrated by PI staining (Fig. S1). We showed that inhibitors lead to a nearly 2-fold reduction in cell invasion (Fig. 4A, Tables S3-5). However, we did not find significant differences between the effects of the inhibitors. This may point out that the protein requires functional activity of both the substrate- and nucleotide-binding domains to mediate cell invasion.

To confirm the contribution of mHsp70 to cell migration, we used FACS analysis to separate each primary culture into two subpopulations – with high mHsp70 expression (mHsp70^High^) and with low expression (mHsp70^Low^) (Fig. 4B). An unsorted native cell population (mHsp70^Wt^) was used as a control. Using the automatic imaging system Image ExFluorer (LCI, Namyangju-si, Gyeonggi-do, Korea), we performed intravital cell imaging for 24 h, taking frames every 15 min. The NIS.ai software NIS.ai software allowed us to plot cell movement trajectories characterized by mean speed (µm/h) and straightness. Figure 4C and D shows an example of phase contrast images with trajectories and rose plots with normalized subpopulation tracks. The results of statistical processing showed that the mHsp70^High^ subpopulations (bright green columns) of all the studied primary glioblastoma cell cultures have a higher speed of movement and straightness, compared with the mHsp70^Low^ subpopulations (light green columns) (Fig. 4E, Tables S6-17). For example, the mean speed of GSN subpopulations was 34.27 [33.67; 34.93] µm/h (n_cells_ = 4759) and 18.69 [18.23; 19.18] µm/h (n_cells_ = 3154) for mHsp70^High^ and mHsp70^Low^, respectively (median [95% CI], *p* < 0.0001). Moreover, the mHsp70^High^ subpopulation of ANI that had the highest membrane protein content according to Western blot had also the highest mean speed – 41.85 [40.72; 43.17] (median [95% CI], n_cells_ = 1053), compared with the other subpopulations. As expected, treatment with PES (1 µM) or JG-98 (50 nM) inhibitors resulted in a significant reduction in the mean speed of native primary mHsp70^Wt^ cells (blue columns). However, when using inhibitors in mHsp70^High^, we found that the mean speed increased in two primary cultures (ANI and TMS). This can be explained by the possible presence of a compensatory effect. When part of a cellular Hsp70 is suppressed, the cell initiates mechanisms of protein hyperexpression in order to neutralize the effect of the inhibitor, as a result, the cell begins to migrate more actively. Based on the obtained result, we concluded that higher concentrations of inhibitors are required to reduce the migratory activity of such most aggressive mHsp70-expressing cells.

## Discussion

Malignant brain tumor cells, particularly GBM, have a high invasive ability to penetrate into surrounding healthy tissue. Active cell migration is mediated by the altered functioning of the motor apparatus, affecting both the cytoskeletal system of the cell and various signaling cascades. Targeting the 70 kDa heat shock protein Hsp70 may be an interesting way to suppress the motility of tumor cells. When overexpressed in tumor cells, Hsp70 can function as an oncogene and influence migration, extracellular matrix remodeling, and epithelial-mesenchymal transition [13, 19–23].

In present work, we were interested in whether a membrane-bound Hsp70 could influence the motility of glioblastoma cells. For this we obtained primary cultures of ANI, GSN, IBD, and TMS cells isolated from the intraoperative material of patients with GBM. We confirmed the neuronal and glial nature of the cells, as well as the presence of stemness markers SOX2, Nestin and CD133. This may indicate that primary glioblastoma cells possess a subpopulation of stem cells that exhibit dormant properties. Dormant tumor cells have a more aggressive phenotype, can contribute to the formation of new tumor foci and become activated at a later stage of tumor development, causing local or distant recurrence [24]. The cells were also positive for mHsp70, which, interestingly, was concentrated in lamellipodia and invadopodia region.

To investigate the possible contribution of mHsp70 to the motility of tumor cells, we isolated a membrane fraction enriched in lipid rafts and performed their proteomic analysis. The results revealed the presence of a whole group of chaperones from several families on the membrane of glioblastoma cells. This probably indicates that in tumor cells, membrane-bound chaperones form a tight complex, which allows them to interact with each other, replace the functions they perform, and maintain a pro-tumor phenotype even with targeted inhibition of one of the members of a family [25].

Among the direct interactors of Hsp70, there were a large number of proteins involved in the remodeling of the cytoskeleton and extracellular matrix, cell adhesion and migration, including the small GTPase RhoA. Interestingly, Yi et al. showed that exogenous Hsp70/Hsp70-peptide complex leads to an increase in the rate of migration and invasion of Huh-7 hepatocellular carcinoma cells through activation of the small RhoA GTPase [26]. All this indicates the ability of mHsp70 to regulate the work of identified proteins and influence the migration properties of tumor cells. We assume that in order to ensure the processes of cellular mobility and, possibly, other cell functions, the presence of the entire ensemble of HSPs representatives (i.e. membrane-bound epichaperome) is necessary for the full functioning of the chaperone apparatus on the surface of the plasma membrane. In combination with other chaperones, Hsp70 can stabilize lipid rafts and supports enhanced signaling from raft-associated proteins, which allows cells to realize a highly invasive phenotype.

To confirm our assumptions about the involvement of mHsp70 in GBM cell motility, we sorted each primary culture into two subpopulations mHsp70^High^ and mHsp70^Low^. Using the automatic single-cell imaging system Image Explorer (LCI, Namyangju-si, Gyeonggi-do, Korea) we showed that the presence of mHsp70 on the membrane correlates with the speed of cell movement and the straightness of trajectories. Moreover, the application of small-molecule inhibitors Hsp70 PES and JG-98 led to a decrease not only in cell migration, but also in their invasive potential. Indeed, several recent studies employing mHsp70 targeting peptides, antibodies, and nanoparticles proved the theranostics potency of this approach in preclinical settings [27, 28]. Since radiotherapy and chemotherapy can significantly increase mHsp70 expression [29, 30], the use of combined anti-chaperone therapy may potentially improve treatment efficacy and prolong survival.

However, concentrations of Hsp70 inhibitors significantly but slightly reduced the average rate of glioblastoma cells speed. It is observed that after FACS sorting the initial cell culture into the mHsp70^High^ and mHsp70^Low^ subpopulations, the effect of the inhibitors became less pronounced or did not appear at all. We assume that the applied concentration of inhibitors is insufficient to fully block mHsp70 and a higher concentration of inhibitors is required to suppress such aggressive, rapidly migrating subpopulations of cells.

In our previous study, we evaluated the therapeutic potential of PES and JG-98 inhibitors in in vivo models of intracranial tumors [15]. Wistar rats with C6 glioma, SCID mice with T98G and U251 human glioblastoma were used as a model object. We have shown that both inhibitors effectively increased the overall survival of animals. Moreover, the results of measuring the tumor volume by magnetic resonance volumetry showed that in the experimental groups treated with Hsp70 inhibitors, on the 21st day after cell inoculation, the tumor volume decreased 3-fold as compared to the control group. Furthermore, the effect of Hsp70 inhibitors was demonstrated in preventing the risk of tumor recurrence in in vivo models by uncoupling the Hsp70-HMGB1 complex, which was shown to exert pro-tumor activity [31]. This indicates the promising use of low molecular weight inhibitors as adjunctive therapy for tumors. In summary, herein we have shown that mHsp70 can be involved in the motility of GBM cells. The presence of Hsp70 on the membrane correlates with the migration and invasive activity of cells. Hsp70 is present in lipid rafts in close association with other chaperones families, forming an entire chaperone cluster. Apparently, the active work of such a cluster of proteins on the membrane and its effect on the functioning of their direct interactors can determine the behavior of a cancer cell. All this points to the need for further investigation of the role of protein in the processes of cellular motility using complex proteomic analysis of other HSPs, as well as their interactomic networks. Selective targeting of membrane forms of chaperones from various families can help reduce the migration potential of tumor cells, preventing their active spread and progression of the disease.

## Electronic supplementary material

Below is the link to the electronic supplementary material.

Supplementary Material 1

## Data Availability

No datasets were generated or analysed during the current study.
