# Supplementary material for: Glioblastoma cell motility and invasion is regulated by membrane-associated heat shock protein Hsp70
Source: J Neurooncol. 2025 Jun 24;175(1):255–65. doi: 10.1007/s11060-025-05127-5 (PMC12367852; doi:10.1007/s11060-025-05127-5)
Supplement: Supplementary file 1 — Supplementary Material 1 [file 11060_2025_5127_MOESM1_ESM.pdf]

Supplementary information for

"GLIOBLASTOMA CELL MOTILITY AND INVASION IS REGULATED BY MEMBRANE-ASSOCIATED HEAT SHOCK PROTEIN HSP70"

Ruslana Likhomanova[1,2#], Elena Oganesyan[2#], Natalia Yudintceva[1,2], Georgii Fofanov[2], Anastasiya Nechaeva[2], Alexei Ulitin[3], Aleksander Kim[2], Nikolay Aksenov[1], Alla Shatrova[1], Rustam Ziganshin[4], Danila Bobkov[1,2], Konstantin Samochernykh[2,3], Stephanie E. Combs[4], Maxim Shevtsov[1,2,5]

[1]Institute of Cytology of the Russian Academy of Sciences (RAS), St. Petersburg, Russia.
[2]Personalized Medicine Centre, Almazov National Medical Research Centre, St. Petersburg, Russia.
[3]Polenov Neurosurgical Institute, Almazov National Medical Research Centre, St. Petersburg, Russia;
[4]Shemyakin-Ovchinnikov Institute of Bioorganic Chemistry Russian Academy of Sciences (RAS), Moscow, Russia.
[5]Klinikum Rechts der Isar, Technical University of Munich, Munich, Germany.

#R. Likhomanova and E. Oganesyan contributed equally to this article.
Corresponding Author: Prof. Dr. Maxim Shevtsov, M.D., Ph.D., Klinikum rechts der Isar, Technical University of Munich, Ismaninger Street. 22, Munich 81675, Germany. E-mail: maxim.shevtsov@tum.de Tel.: +491731488882

Table S1. Characteristics of patients with High-Grade Gliomas.

**Patient code**	**Age (years)**	**Sex**	**KPS before surgery**	**Dexamethasone before surgery (mg)**	**Tumor site (lobe)**	**Hemi-sphere**	**Histology**	**MIB-1 index (%)**	**FGS**
Adult patients									
ANI	57	male	70	12	frontal lobe	left	GB IDH-wt	20	no
GSN	44	male	60	12	frontal lobe	right	GB IDH-wt	35	no
Pediatric patients									
IBD	11	male	70	1	thalamus	left	pHGG	20	no
TMS	3	female	70	1	temporal	left	pHGG	70	yes

Notes: KPS – Karnofsky Performance Scale; GB – glioblastoma; wt – wildtype; pHGG – Diffuse paediatric-type high-grade glioma; FGS – Fluorescence-Guided Surgery with 5-aminolevulinic acid.

Table S2. The quantitative content (%) of $mHsp70^{High}$ and $mHsp70^{Low}$ subpopulations of primary glioblastoma cells based on gating by FACS.

**Primary cells**	**$mHsp70^{High}$, %**	**$mHsp70^{Low}$, %**
ANI	8.0 ± 4.6	18.3 ± 12.9
GSN	16.5 ± 2.1	17.0 ± 2.8
IBD	16.3 ± 4.9	18.8 ± 13.3
TMS	19.7 ± 1.5	21.0 ± 3.5

Table S3. Median ± 95% confidence interval of invaded ANI, GSN, IBD, TMS cell (%) in control group and treated with 1 µM PES or 50 nM JG-98.

**Cell culture**	**Group**	**N_{images}**	**Median ± [95% CI]**
**ANI**	Control	60	90.00 [75.00; 105.0]
	+PES	60	60.00 [45.00; 75.0]
	+JG-98	60	45.00 [30.00; 60.0]
**GSN**	Control	60	83.17 [71.29; 106.9]
	+PES	59	35.64 [35.64; 47.53]
	+JG-98	60	59.41 [47.53; 71.29]
**IBD**	Control	59	73.68 [50.00; 136.8]
	+PES	60	50.00 [36.84; 71.05]
	+JG-98	60	47.37 [31.58; 78.95]
**TMS**	Control	60	97.25 [68.07; 107.0]
	+PES	60	48.62 [38.90; 68.07]
	+JG-98	60	38.90 [29.17; 48.62]

Table S4. Tests for normal distribution for invaded ANI, GSN, IBD, TMS cell (%) in control group and treated with 1 µM PES or 50 nM JG-98. P significance levels: * $p < 0.05$; ** $p < 0.01$; *** $p < 0.001$; ****$p < 0.0001$; ns, not significant.

**Cell culture**	**Group**	**Shapiro-Wilk test**		**Kolmogorov-Smirnov test**	
		**W**	**P value**	**KS distance**	**P value**
**ANI**	Control	0.8692	< 0.0001****	0.1325	0.0105*
	+PES	0.9460	0.0101*	0.1263	0.0185*
	+JG-98	0.8807	< 0.0001****	0.1630	0.0004***
**GSN**	Control	0.9190	0.0007***	0.1514	0.0016**
	+PES	0.8452	< 0.0001****	0.1970	< 0.0001****
	+JG-98	0.9261	0.0014**	0.1450	0.0031**
**IBD**	Control	0.9057	0.0002***	0.1574	0.0009***
	+PES	0.9349	0.0032**	0.1472	0.0024**
	+JG-98	0.9101	0.0003***	0.1787	< 0.0001****
**TMS**	Control	0.8972	0.0001***	0.1345	0.0087**
	+PES	0.8844	< 0.0001****	0.1906	< 0.0001****
	+JG-98	0.8621	< 0.0001****	0.2166	< 0.0001****

Table S5. Wilcoxon rank sum test for multiple pairwise comparison of invaded ANI, GSN, IBD, TMS cell (%) in control group and treated with 1 µM PES or 50 nM JG-98. P significance levels: * p < 0.05; ** p < 0.01; *** p < 0.001; ****p < 0.0001; ns, not significant.

	**Group 1**	**Group 2**	**P value**	**P signif.**
**Invaded cells (%)**	ANI Control	ANI +PES	< 0.0001	****
	ANI Control	ANI +JG-98	< 0.0001	****
	GSN Control	GSN +PES	< 0.0001	****
	GSN Control	GSN +JG-98	0.0001	***
	IBD Control	IBD +PES	0.0140	*
	IBD Control	IBD +JG-98	0.0186	*
	TMS Control	TMS +PES	< 0.0001	****
	TMS Control	TMS +JG-98	< 0.0001	****

Table S6. Median ± 95% confidence interval of cell track parameters of ANI cells + 1 µM PES or 50 nM JG-98 inhibitors.

**Parameter**	**mHsp70^{Wt}**			**mHsp70^{Low}**			**mHsp70^{High}**		
	**Control (N=3998, Median ± [95% CI])**	**+PES (N=3971, Median ± [95% CI])**	**+JG-98 (N=4000, Median ± [95% CI])**	**Control (N=1564, Median ± [95% CI])**	**+PES (N=1549, Median ± [95% CI])**	**+JG-98 (N=1513, Median ± [95% CI])**	**Control (N=1053, Median ± [95% CI])**	**+PES (N=965, Median ± [95% CI])**	**+JG-98 (N=1019, Median ± [95% CI])**
**Mean speed, µm/h**	29.51 [29.03; 30.06]	27.17 [26.65; 27.80]	21.08 [20.58; 21.57]	34.92 [32.71; 35.13]	33.84 [32.71; 35.13]	33.81 [32.86; 34.79]	41.85 [40.72; 43.17]	42.96 [41.40; 44.93]	44.79 [43.30; 46.37]
**Straightness**	0.3695 [0.3614; 0.3769]	0.3504 [0.3419; 0.3585]	0.3221 [0.3141; 0.3289]	0.2290 [0.2198; 0.2374]	0.2050 [0.1939; 0.2149]	0.2282 [0.2157; 0.2372]	0.2671 [0.2503; 0.2925]	0.2843 [0.2690; 0.3062]	0.2847 [0.2686; 0.3002]

Table S7. Tests for normal distribution for tracks mean speed and straightness in different experimental groups of ANI cells + 1 µM PES or 50 nM JG-98 inhibitors experiment data. P significance levels: * p < 0.05; ** p < 0.01; *** p < 0.001; ****p < 0.0001; ns, not significant.

		**Shapiro-Wilk test**		**Kolmogorov-Smirnov test**	
		**W**	**P value**	**KS distance**	**P value**
**Mean speed, µm/h**	mHsp70^{Wt} Control	0.9821	< 0.0001****	0.03169	< 0.0001****
	mHsp70^{Wt} +PES	0.9746	< 0.0001****	0.03813	< 0.0001****
	mHsp70^{Wt} +JG-98	0.9662	< 0.0001****	0.05631	< 0.0001****
	mHsp70^{Low} Control	0.9762	< 0.0001****	0.05437	< 0.0001****
	mHsp70^{Low} +PES	0.9740	< 0.0001****	0.05865	< 0.0001****

	mHsp70^{Low} +JG-98	0.9764	< 0.0001****	0.07038	< 0.0001****
	mHsp70^{High} Control	0.9787	< 0.0001****	0.05605	< 0.0001****
	mHsp70^{High} +PES	0.9672	< 0.0001****	0.06163	< 0.0001****
	mHsp70^{High} +JG-98	0.9634	< 0.0001****	0.07002	< 0.0001****
**Straightness**	mHsp70^{Wt} Control	0.9771	< 0.0001****	0.08730	< 0.0001****
	mHsp70^{Wt} +PES	0.9853	< 0.0001****	0.08638	< 0.0001****
	mHsp70^{Wt} +JG-98	0.9728	< 0.0001****	0.09655	< 0.0001****
	mHsp70^{Low} Control	0.9505	< 0.0001****	0.07030	< 0.0001****
	mHsp70^{Low} +PES	0.9386	< 0.0001****	0.09258	< 0.0001****
	mHsp70^{Low} +JG-98	0.9354	< 0.0001****	0.08770	< 0.0001****
	mHsp70^{High} Control	0.9404	< 0.0001****	0.1019	< 0.0001****
	mHsp70^{High} +PES	0.9369	< 0.0001****	0.09364	< 0.0001****
	mHsp70^{High} +JG-98	0.9484	< 0.0001****	0.07925	< 0.0001****

Table S8. Wilcoxon rank sum test for multiple pairwise comparison of migration parameters of ANI cells + 1 μM PES or 50 nM JG-98 inhibitors experimental data groups. P significance levels: * $p < 0.05$; ** $p < 0.01$; *** $p < 0.001$; ****$p < 0.0001$; ns, not significant.

	**Group 1**	**Group 2**	**P value**	**P signif.**
**Mean speed, µm/h**	mHsp70^{Wt} Control	mHsp70^{Wt} +PES	< 0.0001	****
	mHsp70^{Wt} Control	mHsp70^{Wt} +JG-98	< 0.0001	****
	mHsp70^{Low} Control	mHsp70^{Low} +PES	0.0539	ns
	mHsp70^{Low} Control	mHsp70^{Low} +JG-98	0.1869	ns
	mHsp70^{High} Control	mHsp70^{High} +PES	0.2026	ns
	mHsp70^{High} Control	mHsp70^{High} +JG-98	0.0233	*
	mHsp70^{High} Control	mHsp70^{Low} Control	< 0.0001	****
**Straightness**	mHsp70^{Wt} Control	mHsp70^{Wt} +PES	< 0.0001	****
	mHsp70^{Wt} Control	mHsp70^{Wt} +JG-98	< 0.0001	****
	mHsp70^{Low} Control	mHsp70^{Low} +PES	< 0.0001	****
	mHsp70^{Low} Control	mHsp70^{Low} +JG-98	0.7203	ns
	mHsp70^{High} Control	mHsp70^{High} +PES	0.4113	ns

	mHsp70^{High} Control	mHsp70^{High} +JG-98	0.5875	ns
	mHsp70^{High} Control	mHsp70^{Low} Control	< 0.0001	****

Table S9. Median ± 95% confidence interval of cell track parameters of GSN cells + 1 μM PES or 50 nM JG-98 inhibitors.

**Parameter**	**mHsp70^{Wt}**			**mHsp70^{Low}**			**mHsp70^{High}**		
	**Control (N=3327, Median ± [95% CI])**	**+PES (N=2670, Median ± [95% CI])**	**+JG-98 (N=3571, Median ± [95% CI])**	**Control (N=7459, Median ± [95% CI])**	**+PES (N=4934, Median ± [95% CI])**	**+JG-98 (N=5948, Median ± [95% CI])**	**Control (N=3154, Median ± [95% CI])**	**+PES (N=2584, Median ± [95% CI])**	**+JG-98 (N=3274, Median ± [95% CI])**
**Mean speed, μm/h**	31.93 [31.32; 32.36]	29.97 [29.43; 30.50]	29.32 [28.74; 29.80]	18.69 [18.23; 19.18]	19.33 [18.97; 19.77]	15.18 [14.84; 15.48]	34.27 [33.67; 34.93]	33.53 [32.71; 34.51]	30.16 [29.55; 30.89]
**Straightness**	0.1818 [0.1753; 0.1872]	0.1700 [0.1637; 0.1761]	0.1593 [0.1551; 0.1634]	0.1754 [0.1646; 0.1839]	0.1402 [0.1312; 0.1498]	0.1268 [0.1199; 0.1243]	0.1928 [0.1867; 0.1981]	0.1759 [0.1700; 0.1816]	0.1795 [0.1751; 0.1856]

Table S10. Tests for normal distribution for tracks mean speed and straightness in different experimental groups of GSN cells + 1 μM PES or 50 nM JG-98 inhibitors experiment data. P significance levels: * p < 0.05; ** p < 0.01; *** p < 0.001; ****p < 0.0001; ns, not significant.

		**Shapiro-Wilk test**		**Kolmogorov-Smirnov test**	
		**W**	**P value**	**KS distance**	**P value**
**Mean speed, μm/h**	mHsp70^{Wt} Control	0.9872	< 0.0001****	0.03537	< 0.0001****
	mHsp70^{Wt} +PES	0.9843	< 0.0001****	0.04033	< 0.0001****
	mHsp70^{Wt} +JG-98	0.9861	< 0.0001****	0.03314	< 0.0001****
	mHsp70^{Low} Control	0.9526	< 0.0001****	0.07749	< 0.0001****
	mHsp70^{Low} +PES	0.9522	< 0.0001****	0.07034	< 0.0001****
	mHsp70^{Low} +JG-98	N too large	-	0.08696	< 0.0001****
	mHsp70^{High} Control	0.9807	< 0.0001****	0.03999	< 0.0001****
	mHsp70^{High} +PES	0.9857	< 0.0001****	0.04310	< 0.0001****
	mHsp70^{High} +JG-98	0.9750	< 0.0001****	0.04225	< 0.0001****
**Straightness**	mHsp70^{Wt} Control	0.9249	< 0.0001****	0.08835	< 0.0001****
	mHsp70^{Wt} +PES	0.9157	< 0.0001****	0.08784	< 0.0001****
	mHsp70^{Wt} +JG-98	0.9143	< 0.0001****	0.09304	< 0.0001****
	mHsp70^{Low} Control	0.9094	< 0.0001****	0.1031	< 0.0001****
	mHsp70^{Low}	0.9131	< 0.0001****	0.09069	< 0.0001****

	+PES				
	mHsp70^{Low} +JG-98	N too large	-	0.1098	< 0.0001****
	mHsp70^{High} Control	0.9261	< 0.0001****	0.08468	< 0.0001****
	mHsp70^{High} +PES	0.9170	< 0.0001****	0.09392	< 0.0001****
	mHsp70^{High} +JG-98	0.9221	< 0.0001****	0.08750	< 0.0001****

Table S11. Wilcoxon rank sum test for multiple pairwise comparison of migration parameters of GSN cells + 1 µM PES or 50 nM JG-98 inhibitors experimental data groups. P significance levels: * $p < 0.05$; ** $p < 0.01$; *** $p < 0.001$; ****$p < 0.0001$; ns, not significant.

	**Group 1**	**Group 2**	**P value**	**P signif.**
**Mean speed, µm/h**	mHsp70^{Wt} Control	mHsp70^{Wt} +PES	< 0.0001	****
	mHsp70^{Wt} Control	mHsp70^{Wt} +JG-98	< 0.0001	****
	mHsp70^{Low} Control	mHsp70^{Low} +PES	0.0489	*
	mHsp70^{Low} Control	mHsp70^{Low} +JG-98	< 0.0001	****
	mHsp70^{High} Control	mHsp70^{High} +PES	0.3841	ns
	mHsp70^{High} Control	mHsp70^{High} +JG-98	< 0.0001	****
	mHsp70^{High} Control	mHsp70^{Low} Control	< 0.0001	****
**Straightness**	mHsp70^{Wt} Control	mHsp70^{Wt} +PES	0.0002	***
	mHsp70^{Wt} Control	mHsp70^{Wt} +JG-98	< 0.0001	****
	mHsp70^{Low} Control	mHsp70^{Low} +PES	< 0.0001	****
	mHsp70^{Low} Control	mHsp70^{Low} +JG-98	< 0.0001	****
	mHsp70^{High} Control	mHsp70^{High} +PES	< 0.0001	****
	mHsp70^{High} Control	mHsp70^{High} +JG-98	0.0001	***
	mHsp70^{High} Control	mHsp70^{Low} Control	0.0001	***

Table S12. Median ± 95% confidence interval of cell track parameters of IBD cells + 1 µM PES or 50 nM JG-98 inhibitors.

**Parameter**	**mHsp70^{Wt}**			**mHsp70^{Low}**			**mHsp70^{High}**		
	**Control (N=1957, Median ± [95% CI])**	**+PES (N=1471, Median ± [95% CI])**	**+JG-98 (N=1797, Median ± [95% CI])**	**Control (N=1105, Median ± [95% CI])**	**+PES (N=1395, Median ± [95% CI])**	**+JG-98 (N=1037, Median ± [95% CI])**	**Control (N=1897, Median ± [95% CI])**	**+PES (N=1829, Median ± [95% CI])**	**+JG-98 (N=1983, Median ± [95% CI])**

**Mean speed, µm/h**	9.847 [9.308; 10.65]	7.814 [7.202; 8.475]	7.125 [6.930; 7.335]	7.550 [7.333; 7.787]	7.474 [7.235; 7.731]	7.298 [6.890; 7.661]	10.89 [10.13; 11.68]	10.74 [9.986; 11.50]	11.56 [10.97; 12.21]
**Straightness**	0.1873 [0.1783; 0.1971]	0.2032 [0.1965; 0.2139]	0.1342 [0.1266; 0.1398]	0.2282 [0.2120; 0.2420]	0.1370 [0.1308; 0.1456]	0.1727 [0.668; 0.1816]	0.2426 [0.2331; 0.2499]	0.2412 [0.2289; 0.2489]	0.2388 [0.2289; 0.2489]

Table S13. Tests for normal distribution for tracks mean speed and straightness in different experimental groups of IBD cells + 1 µM PES or 50 nM JG-98 inhibitors experiment data. P significance levels: * p < 0.05; ** p < 0.01; *** p < 0.001; ****p < 0.0001; ns, not significant.

		**Shapiro-Wilk test**		**Kolmogorov-Smirnov test**	
		**W**	**P value**	**KS distance**	**P value**
**Mean speed, µm/h**	mHsp70^{Wt} Control	0.8937	< 0.0001****	0.1462	< 0.0001****
	mHsp70^{Wt} +PES	0.8800	< 0.0001****	0.1569	< 0.0001****
	mHsp70^{Wt} +JG-98	0.9030	< 0.0001****	0.1403	< 0.0001****
	mHsp70^{Low} Control	0.9139	< 0.0001****	0.1241	< 0.0001****
	mHsp70^{Low} +PES	0.9175	< 0.0001****	0.1201	< 0.0001****
	mHsp70^{Low} +JG-98	0.9004	< 0.0001****	0.1252	< 0.0001****
	mHsp70^{High} Control	0.8993	< 0.0001****	0.1285	< 0.0001****
	mHsp70^{High} +PES	0.9169	< 0.0001****	0.1079	< 0.0001****
	mHsp70^{High} +JG-98	0.9087	< 0.0001****	0.1119	< 0.0001****
**Straightness**	mHsp70^{Wt} Control	0.9401	< 0.0001****	0.08361	< 0.0001****
	mHsp70^{Wt} +PES	0.9450	< 0.0001****	0.07270	< 0.0001****
	mHsp70^{Wt} +JG-98	0.9251	< 0.0001****	0.09735	< 0.0001****
	mHsp70^{Low} Control	0.9309	< 0.0001****	0.08624	< 0.0001****
	mHsp70^{Low} +PES	0.9213	< 0.0001****	0.09448	< 0.0001****
	mHsp70^{Low} +JG-98	0.9360	< 0.0001****	0.08959	< 0.0001****
	mHsp70^{High} Control	0.07962	< 0.0001****	0.07962	< 0.0001****
	mHsp70^{High} +PES	0.9431	< 0.0001****	0.08800	< 0.0001****
	mHsp70^{High} +JG-98	0.9457	< 0.0001****	0.07918	< 0.0001****

Table S14. Wilcoxon rank sum test for multiple pairwise comparison of migration parameters of IBD cells + 1 µM PES or 50 nM JG-98 inhibitors experimental data groups. P significance levels: * p < 0.05; ** p < 0.01; *** p < 0.001; ****p < 0.0001; ns, not significant.

	**Group 1**	**Group 2**	**P value**	**P signif.**
**Mean speed, µm/h**	mHsp70^{Wt} Control	mHsp70^{Wt} +PES	< 0.0001	****

	mHsp70^{Wt} Control	mHsp70^{Wt} +JG-98	< 0.0001	****
	mHsp70^{Low} Control	mHsp70^{Low} +PES	0.8211	ns
	mHsp70^{Low} Control	mHsp70^{Low} +JG-98	0.4302	ns
	mHsp70^{High} Control	mHsp70^{High} +PES	0.0089	**
	mHsp70^{High} Control	mHsp70^{High} +JG-98	0.2558	ns
	mHsp70^{High} Control	mHsp70^{Low} Control	< 0.0001	****
**Straightness**	mHsp70^{Wt} Control	mHsp70^{Wt} +PES	0.0013	**
	mHsp70^{Wt} Control	mHsp70^{Wt} +JG-98	< 0.0001	****
	mHsp70^{Low} Control	mHsp70^{Low} +PES	< 0.0001	****
	mHsp70^{Low} Control	mHsp70^{Low} +JG-98	< 0.0001	****
	mHsp70^{High} Control	mHsp70^{High} +PES	0.5340	ns
	mHsp70^{High} Control	mHsp70^{High} +JG-98	0.7898	ns
	mHsp70^{High} Control	mHsp70^{Low} Control	0.0029	**

Table S15. Median ± 95% confidence interval of cell track parameters of TMS cells + 1 μM PES or 50 nM JG-98 inhibitors.

**Parameter**	**mHsp70^{Wt}**			**mHsp70^{Low}**			**mHsp70^{High}**		
	**Control (N=5754, Median ± [95% CI])**	**+PES (N=2918, Median ± [95% CI])**	**+JG-98 (N=5508, Median ± [95% CI])**	**Control (N=5699, Median ± [95% CI])**	**+PES (N=939, Median ± [95% CI])**	**+JG-98 (N=1128, Median ± [95% CI])**	**Control (N=5669, Median ± [95% CI])**	**+PES (N=734, Median ± [95% CI])**	**+JG-98 (N=862, Median ± [95% CI])**
**Mean speed, μm/h**	27.44 [26.98; 27.89]	23.06 [22.50; 23.68]	24.07 [23.60; 24.49]	17.71 [17.37; 18.09]	15.13 [14.22; 15.89]	17.58 [16.74; 18.47]	24.05 [23.66; 24.42]	25.51 [24.45; 26.33]	24.68 [23.90; 25.56]
**Straightness**	0.1929 [0.1858; 0.2002]	0.2488 [0.2378; 0.2586]	0.2458 [0.2401; 0.2540]	0.2002 [0.1921; 0.2073]	0.2049 [0.1914; 0.2212]	0.2420 [0.2263; 0.2609]	0.2509 [0.2435; 0.2576]	0.2152 [0.2036; 0.2311]	0.1830 [0.1705; 0.1920]

Table S16. Tests for normal distribution for tracks mean speed and straightness in different experimental groups of TMS cells + 1 μM PES or 50 nM JG-98 inhibitors experiment data. P significance levels: * p < 0.05; ** p < 0.01; *** p < 0.001; ****p < 0.0001; ns, not significant.

		**Shapiro-Wilk test**		**Kolmogorov-Smirnov test**	
		**W**	**P value**	**KS distance**	**P value**
**Mean speed, μm/h**	mHsp70^{Wt} Control	N too large	-	0.04895	< 0.0001****
	mHsp70^{Wt} +PES	0.9743	< 0.0001****	0.05472	< 0.0001****
	mHsp70^{Wt}	N too large	-	0.06031	< 0.0001****

	+JG-98				
	mHsp70^{Low} Control	N too large	-	0.06501	< 0.0001****
	mHsp70^{Low} +PES	0.9606	< 0.0001****	0.06272	< 0.0001****
	mHsp70^{Low} +JG-98	0.9609	< 0.0001****	0.05543	< 0.0001****
	mHsp70^{High} Control	N too large	-	0.05754	< 0.0001****
	mHsp70^{High} +PES	0.9679	< 0.0001****	0.06625	< 0.0001****
	mHsp70^{High} +JG-98	0.9780	< 0.0001****	0.06584	< 0.0001****
**Straightness**	mHsp70^{Wt} Control	N too large	-	0.06842	< 0.0001****
	mHsp70^{Wt} +PES	0.9473	< 0.0001****	0.08094	< 0.0001****
	mHsp70^{Wt} +JG-98	N too large	-	0.08137	< 0.0001****
	mHsp70^{Low} Control	N too large	-	0.1150	< 0.0001****
	mHsp70^{Low} +PES	0.9350	< 0.0001****	0.09691	< 0.0001****
	mHsp70^{Low} +JG-98	0.9357	< 0.0001****	0.08733	< 0.0001****
	mHsp70^{High} Control	N too large	-	0.1122	< 0.0001****
	mHsp70^{High} +PES	0.9436	< 0.0001****	0.08750	< 0.0001****
	mHsp70^{High} +JG-98	0.9449	< 0.0001****	0.08522	< 0.0001****

Table S17. Wilcoxon rank sum test for multiple pairwise comparison of migration parameters of TMS cells + 1 µM PES or 50 nM JG-98 inhibitors experimental data groups. P significance levels: * $p < 0.05$; ** $p < 0.01$; *** $p < 0.001$; ****$p < 0.0001$; ns, not significant.

	**Group 1**	**Group 2**	**P value**	**P signif.**
**Mean speed, µm/h**	mHsp70^{Wt} Control	mHsp70^{Wt} +PES	< 0.0001	****
	mHsp70^{Wt} Control	mHsp70^{Wt} +JG-98	< 0.0001	****
	mHsp70^{Low} Control	mHsp70^{Low} +PES	< 0.0001	****
	mHsp70^{Low} Control	mHsp70^{Low} +JG-98	0.4544	ns
	mHsp70^{High} Control	mHsp70^{High} +PES	0.0004	***
	mHsp70^{High} Control	mHsp70^{High} +JG-98	0.0121	*
	mHsp70^{High} Control	mHsp70^{Low} Control	< 0.0001	****
**Straightness**	mHsp70^{Wt} Control	mHsp70^{Wt} +PES	< 0.0001	****
	mHsp70^{Wt} Control	mHsp70^{Wt} +JG-98	< 0.0001	****
	mHsp70^{Low}	mHsp70^{Low}	0.8656	ns

	Control	+PES		
	mHsp70^{Low} Control	mHsp70^{Low} +JG-98	< 0.0001	****
	mHsp70^{High} Control	mHsp70^{High} +PES	< 0.0001	****
	mHsp70^{High} Control	mHsp70^{High} +JG-98	< 0.0001	****
	mHsp70^{High} Control	mHsp70^{Low} Control	< 0.0001	****

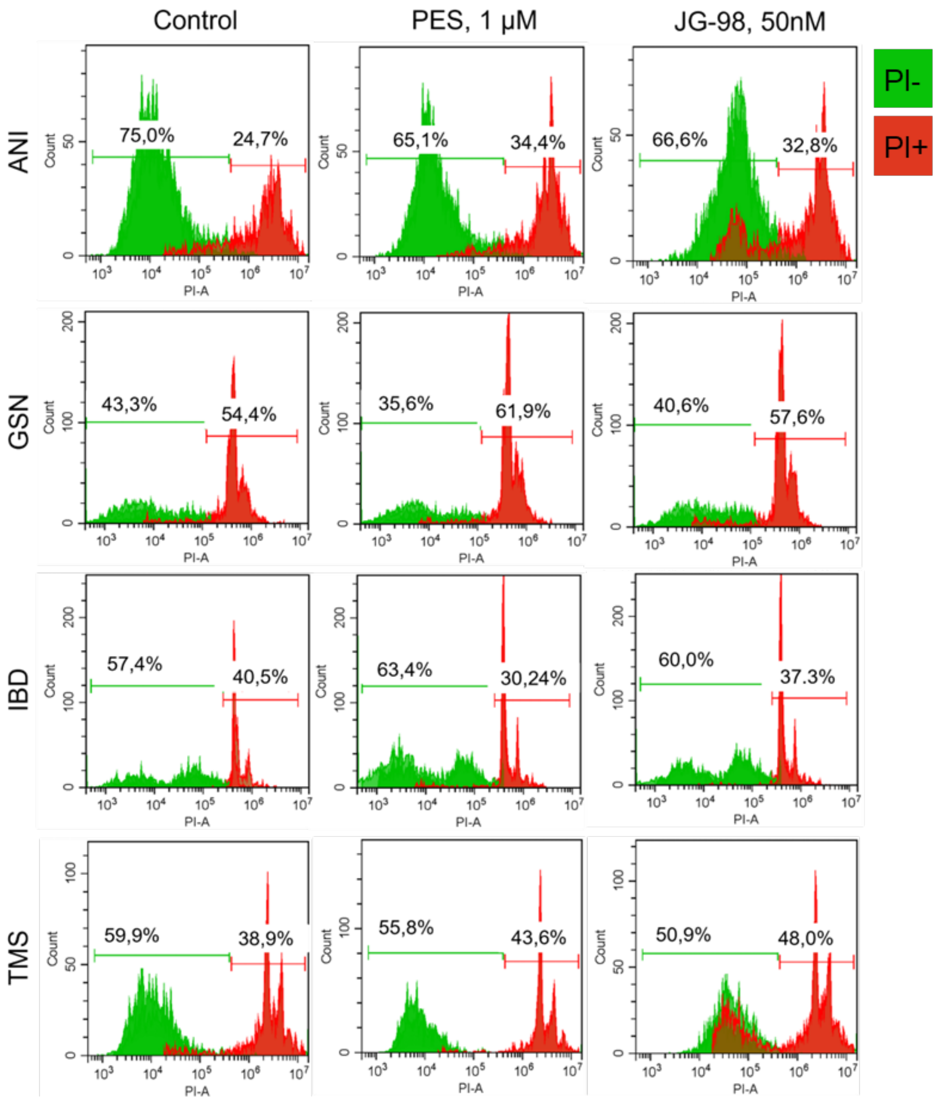

Figure S1. Hsp70 inhibitors PES and JG-98 at concentrations of 1 μM and 50 nM, respectively, do not affect the viability of primary glioblastoma cells. Flow cytometry analysis of cells stained with PI. Green color indicates living cells (PI-), red color indicates dead cells (PI+).

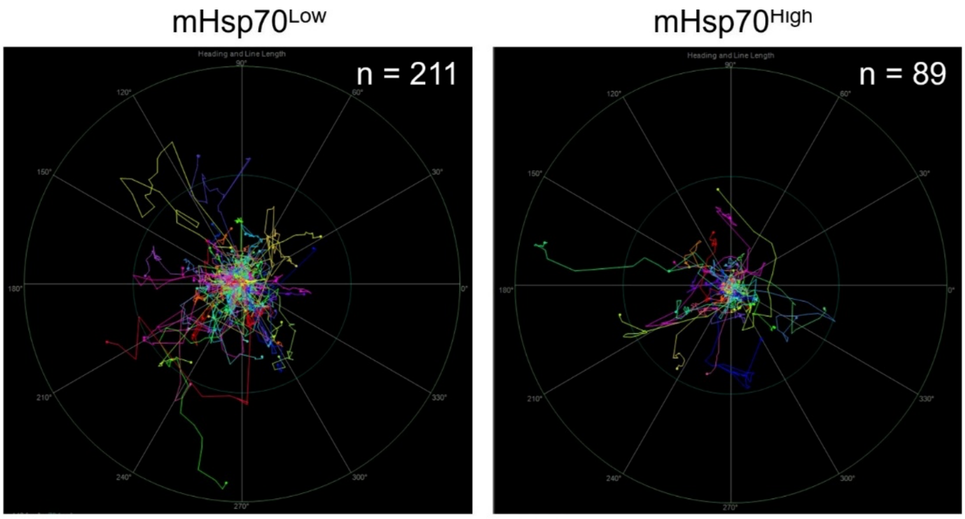

Figure S2. Example of normalized tracks (rose plots) of mHsp70^{Low} and mHsp70^{High} subpopulations of IBD cells.
